# Collaboration Between Public Heath and Law Enforcement: The Constitutional Challenge

**DOI:** 10.3201/eid0810.020465

**Published:** 2002-10

**Authors:** Edward P. Richards

**Affiliations:** * Louisiana State University, Baton Rouge, Louisiana, USA

**Keywords:** police power, anthrax, quarantine, bioterrorism response, *Bacillus anthracis*, law, criminal investigation, Federal Bureau of Investigation

In their article “Collaboration Between Public Heath and Law Enforcement: New Paradigms and Partnerships for Bioterrorism Planning and Response,” Butler et al. present a valuable introduction to the practical problems of coordinating public health and criminal law investigations (1). While this problem is not new in public health, the events of September 11, 2001, have given it a special urgency. This commentary outlines the constitutional constraints on such collaborations, with the objective of helping public health and law-enforcement personnel resolve issues that are not addressed by the article.

## Constitutional Limitations

The Constitution is the source of all legal authority in the United States. Written in 1789, the Constitution was shaped by the events of the time. The weak union of the Articles of Confederation made it difficult to wage the Revolutionary War, so the Constitution provided a strong central government with the power to wage war and raise revenue directly, without depending on state legislatures. The abuses of power by English colonial governors led to the Constitution's Bill of Rights, which strictly limits the state's powers to prosecute and punish individuals for violating the laws—the criminal law power—and to seize personal property for governmental use—the takings power.

At the same time, the terrible toll exacted on the colonies by epidemic disease (2) led the drafters to allow the states very broad powers to abate nuisances and regulate other threats to the public health. The public health authority is known as the police powers (3), as in "to police," meaning to clean up. Formal law-enforcement departments were formed several years after the ratification of the Constitution. Originally, these departments had broader responsibilities than do modern law-enforcement agencies, including some public health functions, so calling them police forces was more consistent with their original function than their current one.

Under the criminal law power, persons who are accused of crimes 1) may not be subjected to search and seizures without probable cause; 2) may not be forced to incriminate themselves; 3) are entitled to a jury trial; 4) are entitled to legal counsel if they are indigent; 5) are entitled to have the case against them proved beyond a reasonable doubt; 6) must be prosecuted under a law that clearly identifies the forbidden behavior; and 7) generally have extensive due process rights to assure that they are not improperly imprisoned. Under the takings power, persons whose property is being seized for the public good have the right to a court hearing and to fair market compensation for the property. In contrast, under the police power, public health officials 1) may search and seize without probable-cause warrants; 2) may take enforcement actions without prior court hearings; 3) are entitled to have courts defer to their discretion; 4) have great flexibility in crafting enforcement strategies; and 5) must only prove their cases by a "more probable than not" standard if the actions are challenged in court (4).

From the ratification of the Constitution to the present day, tension has existed between the Bill of Rights and the police powers. In a key precedent case, a health department seized and destroyed 47 barrels of contaminated poultry from a cold storage plant. The owners claimed that they had been denied due process and just compensation for the value of the property. The court ruled that the destruction or regulation of threats to the public health entitled the owner only to minimal due process and no compensation (5). Other cases established that persons who threaten the public health could be quarantined or subjected to other limitations on their liberty without triggering criminal law due process requirements. Recent cases have concerned whether land use regulations that prevent construction are an improper taking (6) and whether closing bathhouses violates the right of free association (7).

From the earliest cases, the courts have recognized that the public health powers, defined too broadly, would undermine the Bill of Rights. The courts demand that the state demonstrate that the action ordered is intended to prevent harm in the future, not to punish for past actions, and that the action is reasonably related to the public health objective. A gonorrhea control program that involved the temporary detention of prostitutes until they could be examined or treated for gonorrhea (8) was found constitutional (9) because the detention was not a punishment and prostitutes were shown to be an important factor in the spread of gonorrhea in the community (10). A fire ordinance that applied only to Chinese-owned laundries was found unconstitutional because it was not rationally related to preventing fires and was thus an impermissible race law (11).

The courts recognize that there is a continuum between pure public health laws and criminal laws. The more closely the action approaches a criminal punishment, the more protection the individual is entitled to. Thus, mental health commitments, which have a public health component but also resemble imprisonment, require more due process protections than does a quarantine order, but fewer protections than a criminal prosecution. Preventive detention of accused criminals, such as mobsters who might kill witnesses, most closely resembles imprisonment for punishment and must be done with almost the same level of due process as a prosecution (12).

The United States Supreme Court and other courts that decide cases based on U.S. Constitutional law give great deference to state laws dealing with communicable disease control and sanitation, the key issues in bioterrorism. Historically, most state courts, construing their own state constitutions, have allowed public health officials the same latitude and discretion for disease control and sanitation as the U.S. Constitution. However, in the 1980s and 1990s, several states revised their communicable disease control laws or passed special AIDS laws that greatly restricted the authority of public health officials. Some of these laws were subsequently revised because they made it impossible to carry out tuberculosis control programs, but many might still interfere with a bioterrorism investigation. While most states allow food inspectors and other sanitary inspectors broad powers in theory, many state laws divide these powers among several agencies, making prompt and effective investigations almost impossible.

## Bioterrorism Investigations

Certain public health functions, such as sexually transmitted diseases (STD) control, have always involved cooperation with the police. In such situations, public health officials usually do not pass on information to the police that would result in the arrest of infected persons for related crimes such as prostitution. Yet even STD clinics report potential child abuse and provide information to law enforcement to assist their investigations. Bioterrorism investigations require close cooperation between public health and law enforcement, which entails some blurring of their usual roles. Public health investigators will function as forensic experts if there is a prosecution, and law enforcement will try to prevent the further spread of disease by identifying and arresting the perpetrators.

Public health officials can respond quickly to an identified threat and can conduct investigations without the limitations of probable-cause warrants. Public health officials cannot use their powers to circumvent the criminal law protections provided by the Constitution (13). Information gained from public health investigations that do not meet criminal due process standards cannot be used in criminal prosecutions, and if such information is relied on by the police, it may contaminate their subsequent investigations and render all their evidence inadmissible.

Information from public health investigations may be used in criminal investigations if two criteria are met. First, the information must be collected and processed with a proper chain of custody so that it can be authenticated by an expert and admitted into evidence. Since careful handling is also critical to proper epidemiologic investigations, this standard of care should be maintained in all investigations. Second, the evidence must be obtained as part of a legitimate public health investigation. For example, food samples taken during an investigation of food poisoning at a picnic could be used in a subsequent criminal trial if the food was found to be intentionally contaminated. In contrast, food inspectors cannot use their authority to inspect a restaurant kitchen as a pretext for searching the lockers of restaurant employees. If evidence were found in an employee's locker, a judge would not be likely to allow it to be admitted in a criminal prosecution of the employee. To be admissible, a law-enforcement officer would need to obtain a search warrant from a judge before searching the lockers. This necessity could delay the search and might raise public health issues if it was feared that a toxic substance was leaking from the locker and endangering the public. Such conflicts between purposes would be much more severe for an agent such as smallpox, for which decontamination to protect the public might destroy all evidence at the site.

From the perspective of law enforcement, all investigations should be done under criminal law standards to ensure that the perpetrators will be punished at the conclusion of the investigation. To a great extent this coordination was possible in the recent anthrax investigations because the event was discovered after the initial exposure and there was no risk of person-to-person spread. In the case of an evolving epidemic of a more communicable agent, it may be necessary to choose between protecting the population and collecting evidence that will be admissible in criminal investigations. Public health and law-enforcement agencies can minimize this potential conflict by careful planning, as outlined in Butler et al. (1). In many states, the public health inspection laws should be harmonized to assure that a team can be quickly assembled with the authority to conduct all necessary inspections, whether they involve restaurants, workplaces, food processing plants, or agribusiness enterprises. By clarifying legal authority before an incident occurs and increasing communication between government agencies, especially between forensic laboratories and the public health laboratories, the necessity to choose between public health and law enforcement can be lessened.
